# Transcriptomics-based investigation of manganese dioxide nanoparticle toxicity in rats’ choroid plexus

**DOI:** 10.1038/s41598-023-35341-y

**Published:** 2023-05-25

**Authors:** Chun-Yan Meng, Xin-Yi Ma, Ming-Yan Xu, Sheng-Fei Pei, Yang Liu, Zhuo-Lu Hao, Qing-Zhao Li, Fu-Min Feng

**Affiliations:** 1 School of Public Health, North China University of Science and Technology, Tangshan, Hebei 063210 People’s Republic of China; 2 School of Public Health, Nanjing Medical University, Nanjing, 211166 People’s Republic of China; 3 College of Life Sciences, North China University of Science and Technology, Tangshan, Hebei 063210 People’s Republic of China

**Keywords:** Transcriptomics, Public health

## Abstract

Manganese dioxide nanoparticles (MnO_2_-NPs) have a wide range of applications in biomedicine. Given this widespread usage, it is worth noting that MnO_2_-NPs are definitely toxic, especially to the brain. However, the damage caused by MnO_2_-NPs to the choroid plexus (CP) and to the brain after crossing CP epithelial cells has not been elucidated. Therefore, this study aims to investigate these effects and elucidate potential underlying mechanisms through transcriptomics analysis. To achieve this objective, eighteen SD rats were randomly divided into three groups: the control group (control), low-dose exposure group (low-dose) and high-dose exposure group (high-dose). Animals in the two treated groups were administered with two concentrations of MnO_2_-NPs (200 mg kg^−1^ BW and 400 mg kg^−1^ BW) using a noninvasive intratracheal injection method once a week for three months. Finally, the neural behavior of all the animals was tested using a hot plate tester, open-field test and Y-type electric maze. The morphological characteristics of the CP and hippocampus were observed by H&E stain, and the transcriptome of CP tissues was analysed by transcriptome sequencing. The representative differentially expressed genes were quantified by qRT-PCR. We found that treatment with MnO_2_-NPs could induce learning capacity and memory faculty decline and destroy the structure of hippocampal and CP cells in rats. High doses of MnO_2_-NPs had a more obvious destructive capacity. For transcriptomic analysis, we found that there were significant differences in the numbers and types of differential genes in CP between the low- and high-dose groups compared to the control. Through GO terms and KEGG analysis, high-dose MnO_2_-NPs significantly affected the expression of transporters, ion channel proteins, and ribosomal proteins. There were 17 common differentially expressed genes. Most of them were transporter and binding genes on the cell membrane, and some of them had kinase activity. Three genes, Brinp, Synpr and Crmp1, were selected for qRT-PCR to confirm their expression differences among the three groups. In conclusion, high-dose MnO_2_-NPs exposure induced abnormal neurobehaviour, impaired memory function, destroyed the structure of the CP and changed its transcriptome in rats. The most significant DEGs in the CP were within the transport system.

## Introduction

Although manganese is an essential trace element, excessive exposure can potentially cause nerve damage. Manganese accumulates mostly in the striatum, substantia nigra and globus pallidus, damaging the basal ganglia and leading to abnormal release of dopamine (DA) and to neurodegenerative diseases^1,2^. Manganese can also accumulate in the hippocampus, causing learning dysfunction^3^. The neurotoxic mechanisms of Mn^2+^ include imbalance in cellular ion transport homeostasis, oxidative stress in the endoplasmic reticulum and mitochondria, misfolding of proteins, autophagy and apoptosis^4,5^.

Nanoparticles are emerging as a valuable tool for a wide range of biomedical and instrumental applications and thus have attracted worldwide attention^6,7^. Nevertheless, researchers have confirmed that nanomaterials are not harmless and can affect organisms at the cellular, subcellular and protein levels^8^. The application of manganese oxide nanoparticles (MnOx-NPs) and their derivatives have piqued the interest of researchers worldwide over the years. MnOx-NPs and their derivatives can be widely used in magnetic resonance imaging, biological detection, immunotherapy, tumour therapy and other biomedical fields^9,10^. Prior research has shown that MnO_2_-NPs are toxic to animals and the human body^11,12^. MnO_2_-NPs can enter the brain, causing the accumulation of Mn^2+^ in the brain and inducing nerve injuries^13^, leading to apoptosis and morphological changes in hippocampal cells and inducing Parkinson- like neurobehavioral abnormalities^14^. Li et al. found that MnO_2_-NPs injected into the brains of rats reduced spatial learning ability and changed the functions of dopaminergic neurons and astrocytes^2^. Nonetheless, the mechanism by which MnO_2_-NPs cross the blood–brain barrier (BBB) and blood-cerebrospinal fluid (CSF) barrier (BCSFB) and induce damage to brain tissue remains unclear.

The choroid plexus (CP), which is the main structure that produces cerebrospinal fluid, has an important immune and secretion function^12,15^. It consists of specialized epithelial cells that surround a core of fenestrated capillaries and connective tissues, and is populated by diverse cell types: fibroblasts, macrophages, and dendritic cells. The CP epithelial cells that form the BCSFB are joined by tight junctions^16,17^. Many nanoparticles and modified nanoparticles can enter the brain through the BCSFB by means of passive diffusion, inhibition of effusion, opening of tight junctions, receptor-mediated endocytosis, and adsorption subsumption^18,19^.

RNA sequencing technology provides a favourable experimental means for elucidating various brain diseases. The transcriptome is characterized by plasticity and rapid response to various stimuli. A variety of brain-disease-related models have been studied using this method and have yielded a series of fruitful results^20^. To shed light on how MnO_2_-NPs damage the CP and induce nerve injury, we administered two concentrations of MnO_2_-NPs to rats, performed intratracheal infusion, and observed the damaging effect of MnO_2_-NPs on nerves and the transcriptomic changes in the CP. We observed that MnO_2_-NPs caused injury to CP cells and nerve cells by destroying the transporter system of the CP.

## Materials and methods

### Chemicals

The manganese dioxide nanoparticles (MnO_2_-NPs) used in this study was purchased from Beijing DK nano S&T Ltd (China) and had a spherical morphology, with an average particle size of 50 nm, a purity of 99.9%, and a specific surface area of 30 m^2^ g^−1^. The scanning electron microscope diagram is shown in Supplementary Figure 1. The nanomaterial was prepared with sterile saline at a concentration of 100 mg mL^−1^ and mixed by ultrasonic vibration during use.

### Animal rearing and treatment

Eighteen specific-pathogen-free (SPF) male Sprague Dawley rats, 6–7 weeks old, with body weights (BWs) ranging from 200 to 220 g, were purchased and bred in the Laboratory Animal Center of North China University of Science and Technology. All animals were given ad libitum access to water and food on a 12-h light–dark cycle. All experiments were performed and approved in accordance with the Laboratory Animal Ethics Committee of the North China University of Science and Technology. After adaptive feeding for one week, the animals were randomly divided into three groups, the control group (control), low-dose exposure group (low-dose) and high-dose exposure group (high-dose), with 6 rats in each group.

Inhalation of dust particles is the main mode of occupational manganese exposure^13^, so we chose noninvasive intratracheal injection to simulate this scenario. The experimental treatments were administered by noninvasive intratracheal injection after the rats were anaesthetized with isoflurane (not deep anaesthesia). The volume of injection was determined according to the weight of each animal, but the maximum did not exceed 2 mL. Rats in the low-dose group were exposed to MnO_2_-NPs at a dosage of 200 mg kg^−1^ BW, and those in the high-dose group were exposed to MnO_2_-NPs at a dosage of 400 mg kg^−1^ BW, with those in the control group exposed to an equal volume of physiological saline. All animals were treated once a week for three months.

### Neurobehavioural testing

To comprehensively evaluate neurobehavioural changes in animals, the following tests were designed to assess the sensory abilities, cognitive impairments, learning and memory abilities, and motor abilities of the subjects.

#### Hot plate tester

A hot plate meter was preheated and maintained at a temperature of 55 °C. The time was recorded immediately when rats were placed into the hot plate meter and stopped once the animals appeared the first sign of nociception, paw licking, flinching or jump response. Between every two tests, there was a 30 s rest period took place and during which the plate was cleaned with alcohol. Each animal was tested three times^21^.

#### Open-field test

Rats were individually placed into an open field arena (100 cm × 100 cm × 50 cm). Following previously documented protocols, rat activity was recorded for 3 min with a video camera located above the open field^22,23^. After each test, the open field was thoroughly cleaned with 75% alcohol. In all experiments, rat neurobehaviour was evaluated using the time spent in the centre of the open field, the total number of straddles and the total number of stands. The number of straddles was judged as the number of times that more than 1/2 of the animal's body entered the adjacent square. The number of stands was determined by counting the number of times the rat stood erect on its hind limbs.

#### Y-type electric maze

Rats were subjected to a learning and memory capacity test involving a Y-type electric maze. They were placed beside the Y-type electric maze and allowed to adapt to the environment for 5 min, then testing began randomly starting from one direction. Taking one limb of the Y resulted in rats being shocked with an electrical current (30 V, 0.6 mA). The time needed to escape electrical stimulation to the safe region and the number of the wrong choice were utilized to quantitate learning and memory abilities in the rats. The rats were allowed to rest for 30 s between tests, with 5 min between every 10 tests. The experimental techniques and data analysis procedures were elaborated in reference^24^.

#### Swimming speed

The motor ability of the rats was tested using a water maze device. Each rat was placed at a random starting point in the maze, and the time and distance from the starting point were recorded. The rats' swimming speed was then calculated based on these data^14^.

### Choroid plexus (CP) and hippocampus histology

Three CPs and hippocampi from each group were washed with 1% ice-cold saline and fixed in neutral buffered 10% formalin (Beijing Solarbio Science & Technology Co., Ltd, China). The tissues were embedded in paraffin blocks and then trimmed and sectioned using a microtome to select the lateral ventricle site. Paraffin sections with a thickness of three millimetres were stained with haematoxylin and eosin (H&E)^25,26^.

### mRNA-seq and data analysis

RNA samples were then further purified with magnetic oligo(dT) beads after denaturation. Purified mRNA samples were reverse transcribed into first-strand cDNA, and the second cDNA was further synthesized. Fragmented DNA samples were blunt-ended and adenylated at the 3′ ends. Adaptors were ligated to construct a library. DNA was quantified by Qubit (Invitrogen). After cBot cluster generation, DNA samples were then sequenced by an Illumina HiSeq X Ten SBS instrument from Genergy Bio (Shanghai). Raw data were converted into Fastq format. The number of transcripts in each sample was calculated based on the number of fragments per kilobase of transcript per million fragments mapped (FPKM). Cuffnorm software was used to calculate the FPKM value for each sample, and the values were log2 transformed. DESeq2 software was adopted to calculate the differential gene expression between different samples (v1.16.1, https://bioconductor.org/packages/release/bioc/html/DESeq2.html) For KEGG pathway analysis, the entire set of genes was used as the background list, the differential genes were used as the candidate list, and the P value was calculated. Significant genes were categorized based on gene functions. Databases involved in the study included the Strings (http://string-db.org), KEGG Pathway^27,28^ (http://www.genome.jp/keggbin/show_organism?menu_type=pathway_maps&org=hsa), and UniProt (http://www.uniprot.org/downloads) databases. Wayne diagrams were drawn using the website http://jvenn.toulouse.inra.fr/app/example.html. Figure 5 was drawn by FigDraw (https://www.figdraw.com/static/index.html).

### RNA isolation and quantitative real-time PCR (qRT‒PCR)

The primer sequences are listed in Supplementary Table 1. qRT-PCR was carried out using SYBR Green PCR Master Mix (Beijing Mei5 Biotechnology Co., LTD, China) and a StepOne™ System (Applied Biosystems, China). The amplification system was 20 μL, the amplification conditions were as follows: 95 °C for 2 min for pre-denaturation, followed by 35 cycles of 95 °C for 30 s, 60 °C for 30 s and 72 °C for 30 s for amplification, and 72 °C for 2 min for the holding stage. The relative gene expression levels were calculated using the 2^−ΔΔCt^ method.

### Statistical analysis

SPSS (V19, IBM, America) was used for the statistical analysis of all data by one-way analysis of variance (ANOVA). A p value less than 0.05 (typically ≤ 0.05) was considered to indicate statistical significance.

### Ethical approval

All experiments were performed with consideration for animal welfare and were approved in accordance with Laboratory Animal Ethics Committee of North China University of Science and Technology.

### Statement

The authors declared that experimental animals’ care was in accordance with institutional guidelines. The use of anesthesia for invasive animal experiments was consistent with animal welfare. All experiments complied with the ARRIVE guidelines.

## Results

### MnO_2_-NPs induced neurobehavioural changes and CP injury

**Figure 1:**
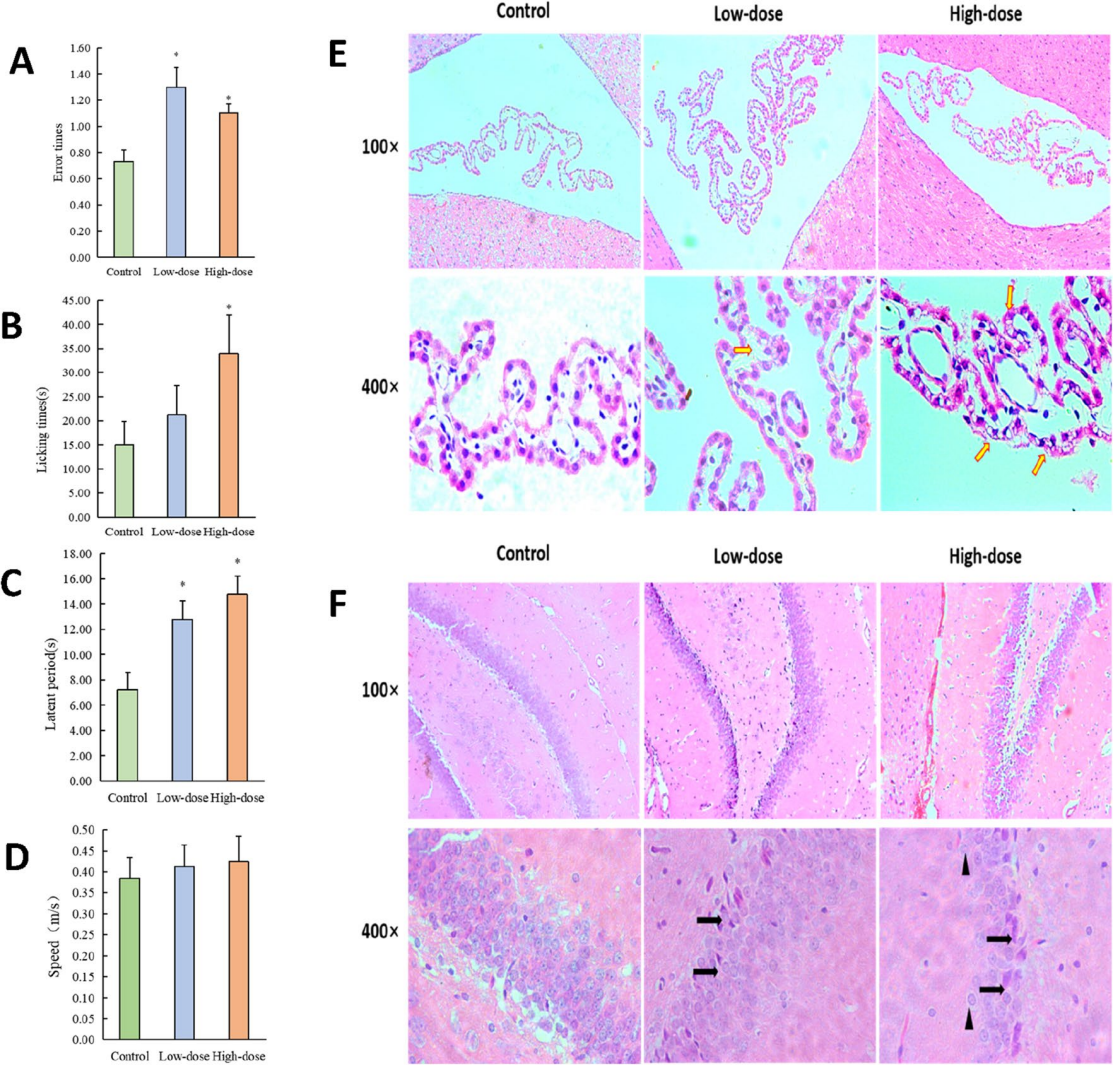
Nano manganese dioxide poisoning caused the decrease in learning and memory and the damage to choroid plexus and hippocampus. (**A**) The number of errors in finding the correct position of the rats in 3 experiments; (**B**) The incubation period of rats in the electric maze experiment; (**C**) The time for rats licking their rear feet; (**D**) Swimming speed of rats in each group. Paraffin sections of CP (**E**) and hippocampus (**F**) stained with H&E dye. In the image (**E**), arrowheads indicate Vesiculated cells of the choroid plexus. In the image (**F**), arrows point to a triangular cell with an unclear nucleus. The blank triangle indicated edema cells. Neurons in hippocampal CA3 region have a disordered arrangement and a reduced hierarchy. The irregular cells became increased, and cell nuclei were hyperchromatic and pyknotic.

Rats were administered MnO_2_-NPs by intratracheal injection for three months. Neurobehavioural testing showed that after exposure, the learning capacity and memory faculty of the rats declined (Fig. 1A, B), and the sensory ability of the hindlimbs dulled (Fig. 1C). However, their swimming speeds showed no changes (Fig. 1D). In the CP epithelium of treated rats, the cell nuclei disappeared, and the cell junctions became loose. It was apparent that in the high-dose group, the structure of the CP was damaged more seriously, the cells were more vacuolated, and the intercellular connections became looser (Fig. 1E). Likewise, MnO_2_-NPs also damaged the hippocampus, causing loose cell connections and irregular arrangements. (Fig. 1F). Neurobehavioural experiments suggested that MnO_2_-NPs had entered the brain tissue and caused cognitive dysfunction, leading to a decline in learning and memory ability in the animals. Furthermore, histomorphological changes suggested that MnO_2_-NPs had caused severe damage to chorioplexus epithelial cells and the hippocampus.

### Selected differentially expressed genes (DEGs) of the CP among the three groups

We harvested CP tissues and analysed the RNA-Seq data from six rats treated with MnO_2_-NP and two rats in the control group (raw mRNA-seq data are shown in Supplementary Tables 2 and 3). A variety of intergroup and intragroup comparisons were conducted using a correlation heatmap (Supplementary Fig. 2).

**Figure 2:**
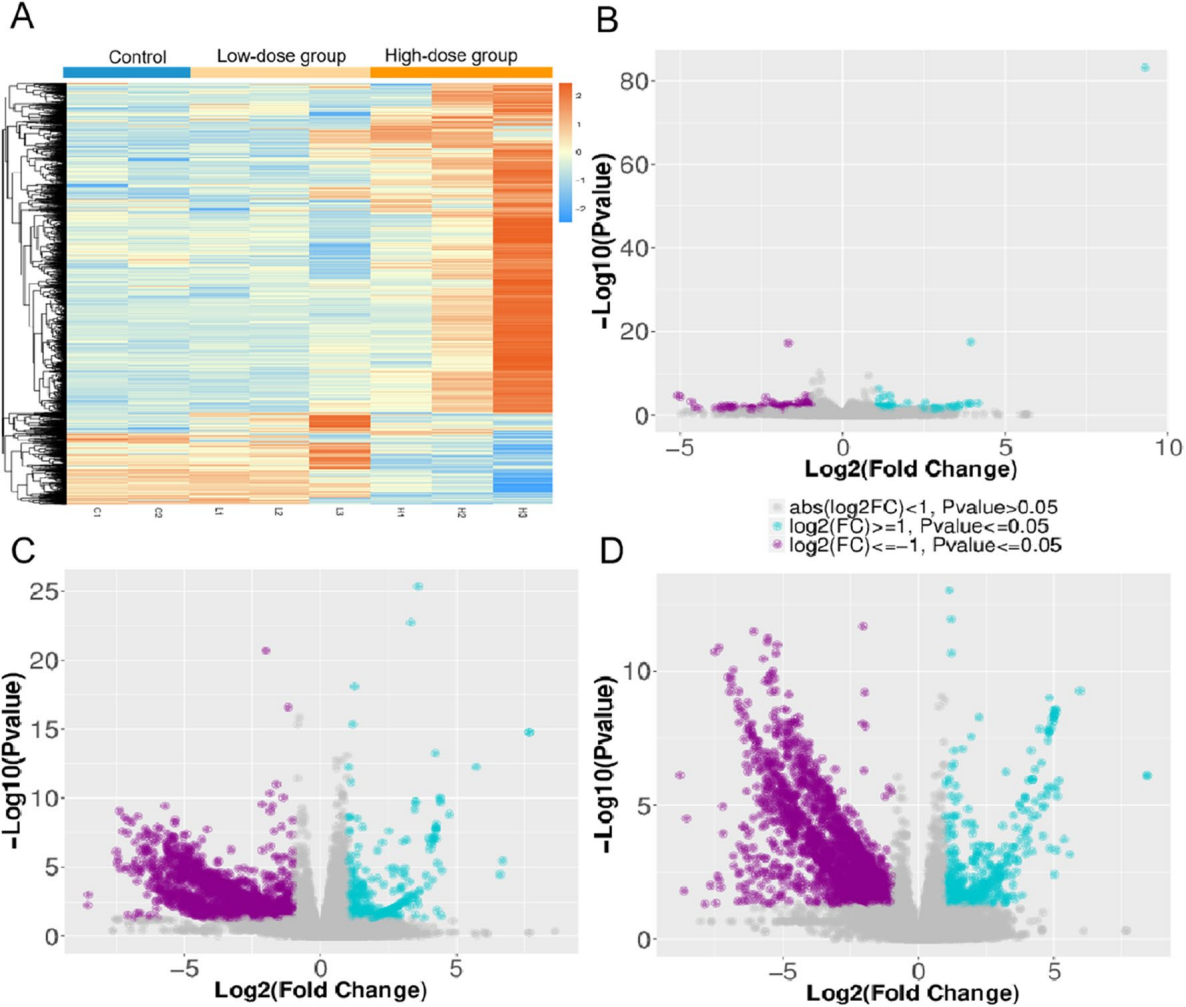
Difference of gene expression (DEGs) between groups. (**A**) Hot map showed regional choroid plexus identity. The cut-off p-value for inclusion of genes was *P* < 0.05. (**B**–**D**) Scatter plot showed DEGs of up- and down-regulation (**B**) low-dose VS control; (**C**) high-dose VS control; (**D**) high-dose VS low-dose.

The logarithmic expression levels of each gene from the six samples are displayed in a heatmap (Fig. 2A) and volcano plot (Fig. 2B–D), facilitating our understanding of the response patterns of gene expression. There was a tremendous difference in gene expression between the control group and the other groups. We chose the screening criteria of *P* < 0.05 and *|log2 (fold change)|* ≥ 1.5. There were 2100 DEGs between the high-dose and low-dose groups, of which 1777 were upregulated and 323 were downregulated; there were 106 DEGs between the low-dose and control groups (71 upregulated and 35 downregulated); There were 1969 DEGs between the high-dose and control groups (1768 upregulated and 201 downregulated). The expression of genes in the CP of rats changed significantly after MnO_2_-NP treatment, and there were more upregulated genes than downregulated genes, especially in the high-dose group. Moreover, the transcriptomic changes between the high-dose and low-dose groups were more significant. Although the toxicity of MnO_2_-NPs in rats showed a specific dose‒response correlation, the DEGs changed more significantly between high-dose and low-dose MnO_2_-NP exposure. The low-dose treatment was 200 mg kg^−1^ of MnO_2_-NPs, which is several times higher than the previously used concentration (equivalent to 11.82 mg Mn kg^−1^)^29^. It is also several times higher than the concentration in mice that showed no apparent toxic effects (35 mg kg^−1^)^30^. Therefore, there may be different mechanisms underlying the damage caused by the two conditions.

### Functional and pathway annotation of DEGs

**Figure 3:**
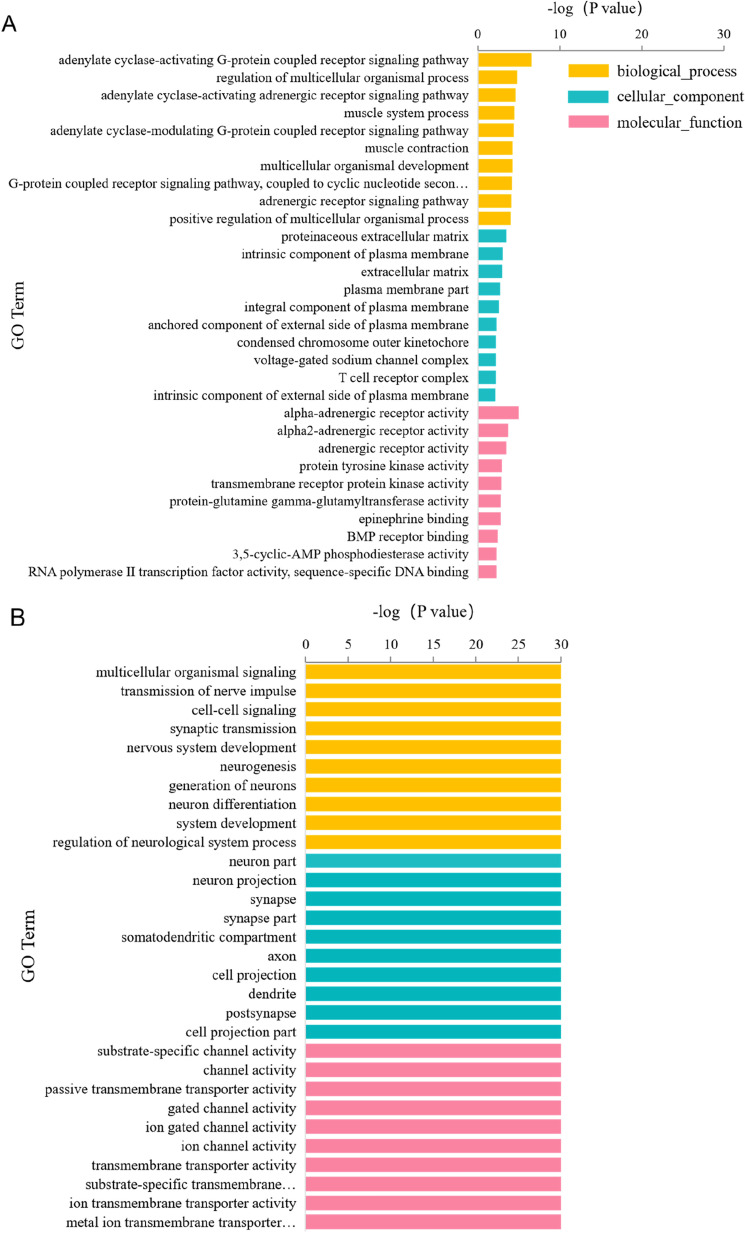

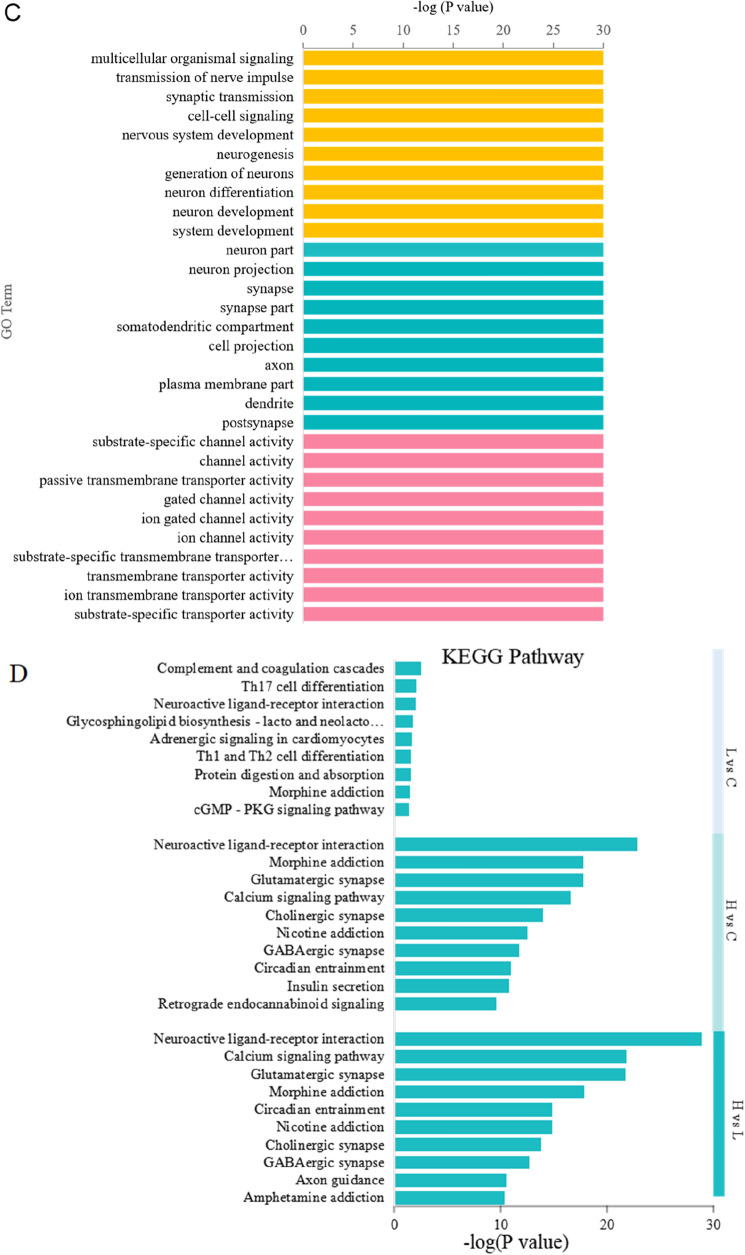
GO enrichment Bar plot display (top GO according to *P* value) and KEGG metabolic pathway enrichment. (**A**) low-dose vs control; (**B**) high-dose vs control; (**C**) high-dose vs low-dose; (**D**) KEGG pathway.

Interestingly, gene expression varied significantly between the groups. A large number of genes showed changes in expression levels, including those for membrane protein receptors, cell communication molecules, transport channels, vesicle transport, kinase proteins and so on. (Supplementary Tables 5–7). In a bar chart of GO term analysis, we ranked the top 10 GO terms of biological process, cellular component and molecular function categories (Fig. 3A–C). Compared with the control group and the low-dose group, the high-dose group showed similar types of DEGs, and fold difference. Together with the fact that we only displayed the top 10 DEGs, this resulted in the −log P values of Fig. 3B and C showing the same bar length. Based on the molecular function GO terms of the DEGs, the genes with the most significant differences between the low-dose group and the control group were adrenergic receptor activity, protein tyrosine kinase, transmembrane receptor protein kinase, and so on. The biological processes clustered mainly in protein kinase signalling pathways. However, between the control and the low-dose group, the DEGs were mainly associated with ion channels, transmembrane transport, synapses, etc. Meanwhile, some biological processes were clustered for communication between nerve cells and neural development.

We ranked the top 40 genes significantly regulated by MnO_2_-NPs in the low-dose and high-dose groups by *P* values. Analysis of the GO molecular function terms suggested that the most differentially expressed genes induced by MnO_2_-NPs were membrane protein receptor, cell communication molecule, transport channel, and kinase function genes. In the high-dose group, RNA-related genes were more strongly downregulated, while channel proteins and kinase receptor proteins were more strongly upregulated (Data are shown in Supplementary Tables 5–7).

Furthermore, KEGG pathway analysis of the DEGs showed that the enriched pathways between the low-dose and control groups were mainly for complement and coagulation cascades, Th17 cell differentiation, adrenergic signalling, glycosphingolipid biosynthesis, and neuroactive ligand-receptor interaction. However, the main enriched pathways between the low- and high-dose groups were neuroactive ligand-receptor interaction, calcium signalling pathway, glutamatergic synapse, etc. (Fig. 3D).

### Different concentrations of MnO_2_-NPs altered gene expression in rats’ CP cells

We analysed the intersection of all the DEGs from the pairwise comparison of the three groups and found 17 common DEGs (*P* < 0.05, *|log2 (fold change)|* ≥ 1.5) (Supplementary Table 4). Most of these common DEGs were transporter and binding genes on the cell membrane, and some of them had kinase activity. Functional annotation of some genes showed that they were also related to neural development and cell cycle, such as *Brinp1* and *Nrarp.*

### DEGs in transport signalling pathways identified by qRT‒PCR

**Figure 4:**
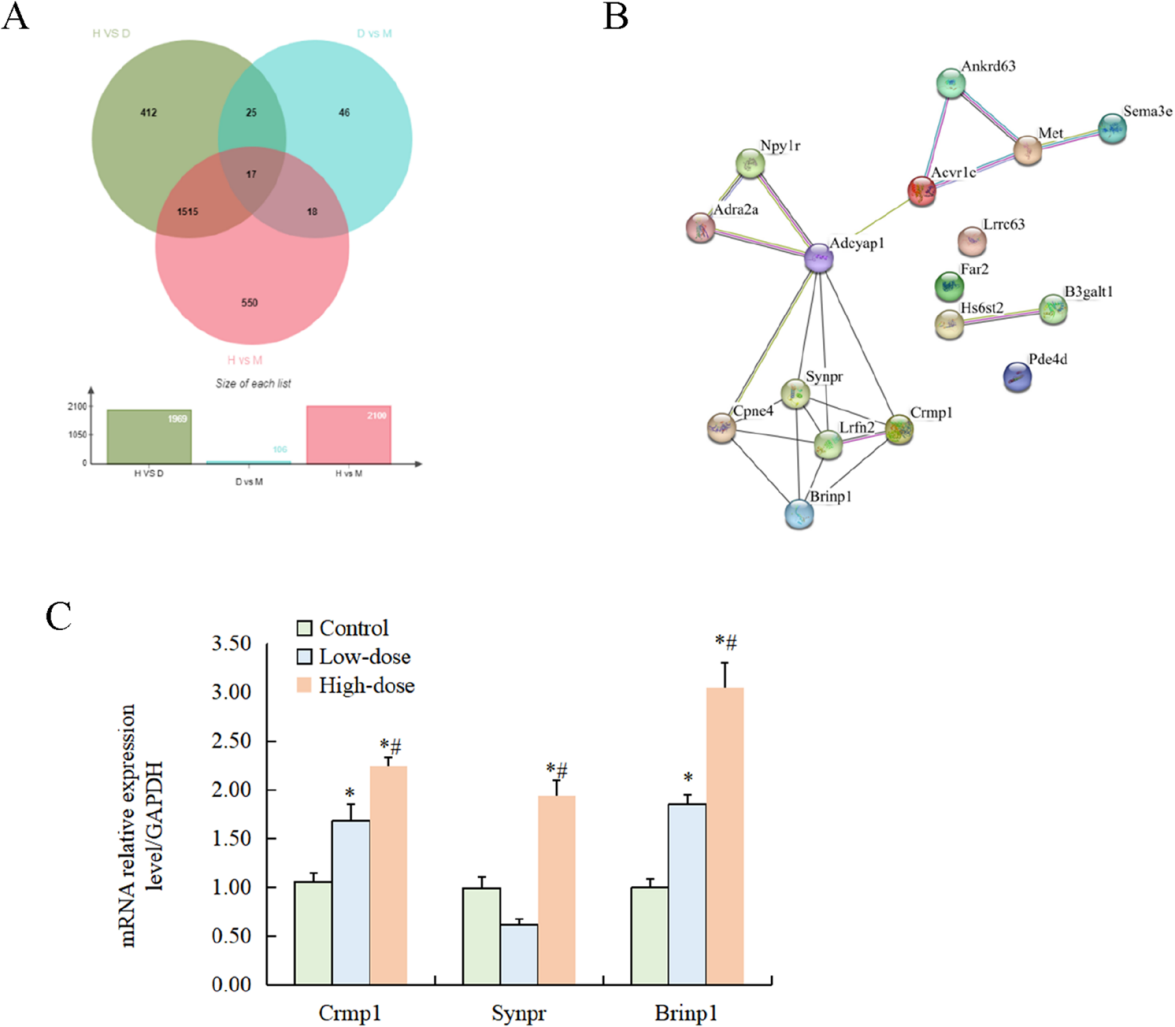
Display and detection of DEGs. (**A**) Venn diagram display of all DEGs. Significant DEGs were selected by *P* < 0.05, *|log2 (fold change)|* ≥ 1.5; (**B**) PPI network of the DEGs. (**C**) Expressions of representative DEGs by QRT-PCR. H, the high-dose group; *L* the low-dose group, *C* the control group; *Indicated compare with the control group *P* < 0.05; ^#^indicated compare with the low-dose group *P* < 0.05.

Furthermore, we used qRT‒PCR to verify the expression levels of three common DEGs among the three groups, *Brinp1* (BMP/retinoic acid inducible neural specific 1), *Synpr* (synaptoporin), and *Crmp1* (collapsin response mediator protein 1). All of these genes were differentially expressed in the CP, and their expression was significantly increased in the high-dose group (Fig. 4).

**Figure 5:**
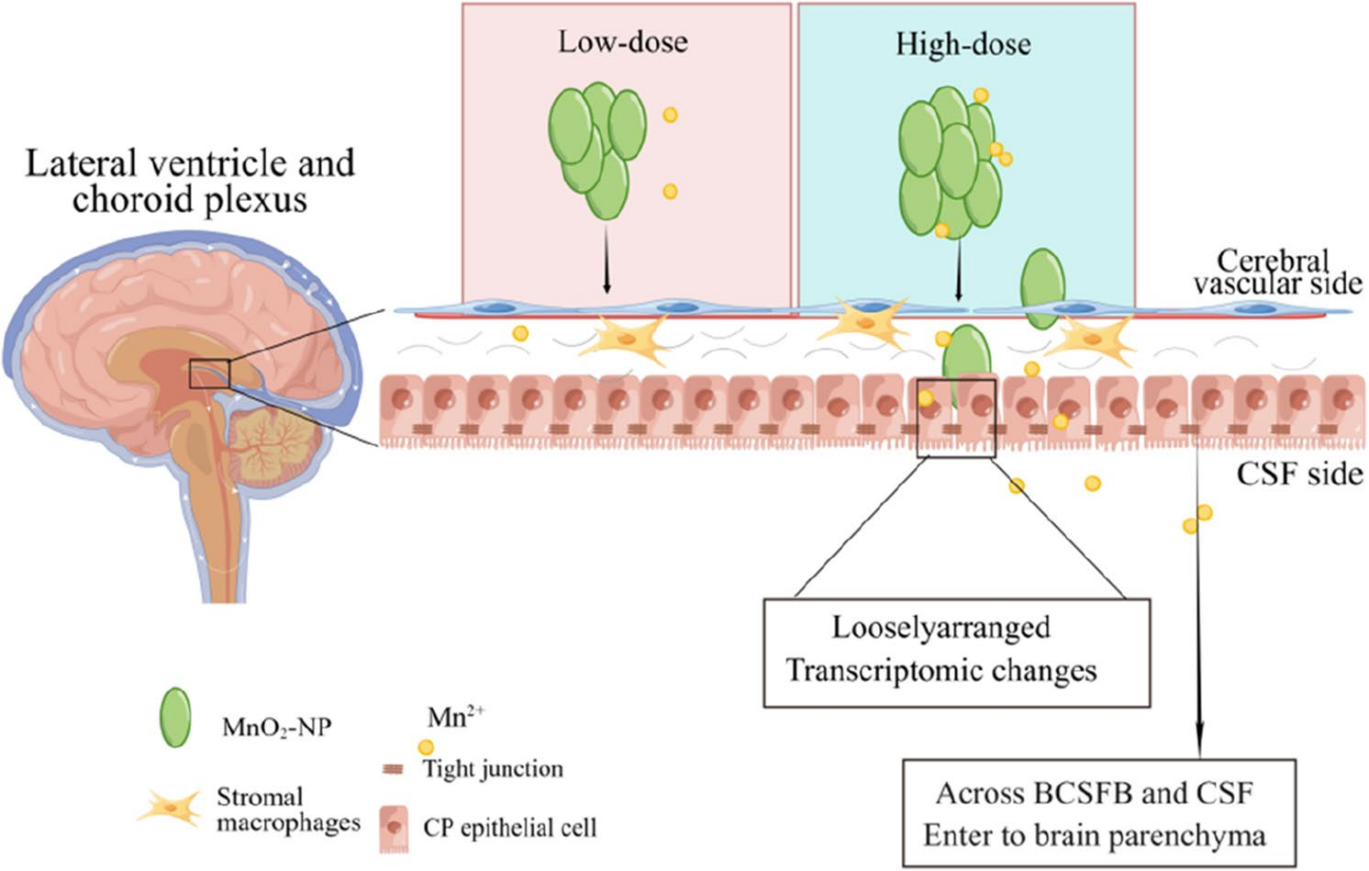
Diagram of the choroid plexus injury induced by MnO_2_-NP. (The diagram was drawn by Figdraw). Nano-manganese oxide will resolve manganese ions in the body, which together with the nanomaterial itself has a toxic effect on the choroid plexus. There are certain differences in the form of damage to the choroid plexus at low and high doses of MnO_2_-NP. Low doses only have a lighter effect on the structure and gene expression of the choroid plexus, while high doses cause obvious changes in the tissue structure of the choroid plexus and changes in gene expression, with the main differential genes concentrated on transporters and tight junction proteins. High doses of nano-manganese make more Mn^2+^ cross the BSCFB and SCF, enter the brain, induce the learning and memory function injury and neurological behavior changes.

In this study, we found many DEGs related to potassium ion channels and cation ion channels. This suggests that there may have been changes in the transport function of the CP and an imbalance in its ion status. In summary, the combined effects may have facilitated the penetration of nanomaterials through the BCSFB to CSF, entering the brain and changing the neurological behaviour of rats. Based on our study, we have proposed a hypothetical mechanism of MnO_2_-NPs, as shown in Fig. 5.

## Discussion

### Determing the dosage

However, there are still limitations in understanding the nanoparticle-mediated toxicity and its exact molecular mechanisms. Studies have suggested that MnO_2_-NPs are safe for human bodies at low doses^31^. They can protect bones and joints from inflammatory damage induced by H_2_O_2_ and can be used as a protective agent because of their reducing properties; the dose used is equivalent to 500 μg kg^−1^^32^. In addition, Mn nanoparticles are widely used as contrast agents in magnetic resonance imaging (MRI) systems^7,9^. A dose of 0.136 mmol Mn kg^−1^ (equivalent to 11.82 mg Mn kg^−1^) was injected into rats, and the permeability of the CP during stroke was shown by MRI^29^. In another study, for MRI angiography, MnO nanoparticles were administered to mice by tail vein injection at a dose equivalent to 5.0 mg Mn kg^−1^^30^. They also concluded that it had no obvious toxic effect on the brains of mice injected intravenously with 35 mg kg^−1^^30^. In contrast, a number of studies have shown that MnO_2_-NPs have toxic effects in vivo and in vitro^2,7,11,26,33^. The CL_50_ of MnO_2_-NP was 0.12 mg L^−1^ in an acute inhalation toxicity test in rats^34^. Synthetic nanomanganese oxides belong to the second class of dangerous chemicals according to the Global Harmonized System of Classification and Labelling of Chemicals (GHS). In an acute oral toxicity test of MnO_2_-NPs at 1000 mg kg^−1^ body weight, pronounced blood damage and DNA damage were observed^26^. Oszlánczi et al. applied 5.26 mg kg^−1^ MnO_2_-NP tracheal infusion once a day, inducing nerve damage in rats^13^.

Thus, there are great differences in the concentrations and modes of administration of MnO_2_-NPs among previous studies, leading to varying conclusions. Given the significant toxic effects, we selected a high dose of 400 mg kg^−1^ body weight and a low dose of 200 mg kg^−1^ body weight for the present study. Our results revealed that the damaging effect of MnO_2_-NPs in the high-dose group was more pronounced than that in the low-dose group.

### Traversing the blood-cerebrospinal fluid barrier

The results of this study showed that MnO_2_-NPs damaged neurological behaviour and learning and memory in rats. However, the effect on swimming and other sports abilities was not significant. Similar to previous studies^2,13,14^, our results indicate that MnO_2_-NPs can enter the brain tissue and damage the CP. However, according to previous studies, there was little difference. Sadeghi et al. reported that intraperitoneal injection of MnO_2_-NPs at 100 µg kg^−1^ can cause Parkinson-like symptoms, hippocampal damage, and changes in motor function^14^. We noted that the MnO_2_-NPs used in their paper had a particle size of 30–60 nm and good dispersion. The nanomaterials used in our study had an average particle size of 50 nm, with poor dispersion observed in the electron microscope images. Regrettably, we did not detect the dispersion of nanomaterials in suspension, which may be one of the key factors affecting the toxicity of nanomaterials^5,7,35^. On the other hand, the destruction of CP may enhance its permeability to inflammatory cells^36^. In KEGG pathway enrichment, we also found Th17 cell differentiation pathway (Fig. 3D), which suggested an inflammatory reaction may occurr in CP cells. Therefore, further experimental verification is needed to determine whether there is any inflammatory injury in the choroid plexus and brain tissue caused by the injury caused by MnO_2_-NPs.

### Disruption of the choroid plexus transport system

The blood-cerebrospinal fluid barrier may be the main route via which manganese enters the brain parenchyma^37^. Mn^2+^ could induce gene expression changes in the CP in rats, as observed through transcriptomic analysis. These DEGs were mainly enriched in the mitochondria, membrane and cytoplasm and participated in the biological processes associated with metabolism and transport pathways^38^. In this study, we found that MnO_2_-NPs induced various changes in biological processes and affected a wide range of related pathways for ion transport and gated channels. Interestingly, the number and types of DEGs at a high concentration (400 mg kg^−1^ body weight) were significantly different from those at a low concentration (200 mg kg^−1^ body weight) (Figs. 2,3). As a selective choroid plexus toxicant, Mn in plasma accumulates in the CP^30,39^. Although it does not directly damage the CP, it can be actively transported to the brain in a unidirectional manner, resulting in serious severe neurotoxicity^37,40^. Our supplementary data have shown the Mn concentration in the CSF, serum and brain cortex (Supplementary Fig. 3). It is likely that Mn accumulated in the CP at low doses has not yet caused damage, whereas at high doses, superfluous Mn began to damage the CP in addition to being transported to brain tissue.

We have displayed the common DEGs between groups in a Wayne chart diagram (Fig. 4A). Most of these genes were associated with ion channels in CP cells, while others were related to neural development, cell cycle, and oxidative stress. Ion channels on the CP play an essential role in maintaining ion concentration between plasma and cerebrospinal fluid, as well as the stability of the CP itself. An imbalance in ion status directly leads to the instability of CP epithelial cells^41^. An experiment on solute Slc39a14 (a gene that uses Mn^2+^ as a substrate for ion transport) knockout mice found that this gene deficiency leads to Mn accumulation in the brain, causing motor deficits in mice^42^. This suggests an important role for Mn transporters.

The considerable degree of variability in sample H3 may have amplified the negative effects of high doses. We discovered that some of the frequently differentially expressed genes were linked to oxidative damage, indicating that nanomanganese dioxide may directly cause oxidative damage to choroid plexus cells. When the dose of nanomanganese dioxide was increased to 400 mg kg^−1^, many receptor proteins and channel protein genes on the membrane of choroid plexus cells were affected, which may have aggravated the passage of manganese ions through BCSFB and caused brain tissue damage.

Another limitation of this study is that additional experiments studying the direct damage of the CP by MnO_2_-NPs and the accumulation of MnO_2_-NPs in the brain were not designed. To address this limitation, more experiments are needed, for example, by directly injecting MnO_2_-NPs into the CP and detecting CP permeability and the distribution of Mn^2+^ in the brain. In addition, in vitro experiments can also be conducted.

### Mechanism of MnO_2_-NP-induced injury

The general mechanism by which metallic oxide nanoparticles induce toxicity is a joint function of the properties of the nanoparticle and its corresponding ability to induce ROS and cause toxicity in cells, genes, and neurons^5^. The neurotoxicity of the NPs can be attributed to oxidative stress triggered by free radical activity. For example, PbO nanoparticles could induce an increase in ROS production in the CP epithelial cell line and break the cell tight connection^43^. MnO_2_-NPs can induce oxidative damage and apoptosis in hippocampal cells^14^.

One theory suggests that metal nanomaterials can release metal ions and interfere with cation transport in cells, ultimately leading to cellular damage. This dissolution process is promoted by the acidic environment of cells^7,44,45^. Mn^2+^ can be dissolved from the surface of MnO_2_-NPs. Meanwhile, nanomaterials can also act as carriers of cations crossing cell membranes and cause mechanical injury^25^. Mn^2+^ damages the basal ganglia, leading to the abnormal release of dopamine (DA) and neurodegenerative diseases^1,44^, and accumulates in the hippocampus, causing learning dysfunction^3^. Previous studies have shown that manganese chloride could induce metabolism and transport protein alterations, which echoes the present study^38,46^. Excessive Mn damages cellular functions by multiple mechanisms, such as oxidative stress, inflammation and protein aggregation^47^. Mn^2+^ can induce a GSH-dependent Fenton reaction in cells^48^. This may also be one of the molecular mechanisms of cell damage caused by MnO_2_-NPs. On the other hand, the stimulation of animal lungs by nanomaterials can cause pulmonary inflammation. Unfortunately, we did not confirm whether the stimulation of inflammatory factors in the blood affects the permeability of the choroid plexus.

Although MnO_2_-NPs have good biocompatibility and have been applied to the diagnosis and treatment of tumors and magnetic resonance imaging^49^, they are not absolutely safe and are neurotoxic beyond a certain dose according to our and previous findings. Studies in occupational medicine have found that manganese exposure in manganese miners is directly related to Parkinson's disease^50^. MnO_2_-NPs tablets could react with strong and weak reducing agents in the biofluid environment, causing potential hazards to the environment and occupational exposure^51,52^. In addition, the potential toxicity of MnO_2_-NPs cannot be ignored due to their ability to consume GSH and cause oxidative damage. However, given the limitations of this study, further investigation is needed to fully elucidate the exact mechanisms underlying the transcriptomic changes induced by MnO_2_-NPs in the CP.

### Supplementary Information

Supplementary Information.

## Data Availability

The datasets generated and/or analyzed during the current study are available in the NCBI repository. The submission number was PRJNA883222 and PRJNA906952.

## References

[1] Balachandran RC (2020). Brain manganese and the balance between essential roles and neurotoxicity. J. Biol. Chem..

[2] Li T (2014). Effects of Nano-MnO_2_ on dopaminergic neurons and the spatial learning capability of rats. Int. J. Environ. Res. Public Health.

[3] Lee EY (2019). Higher hippocampal mean diffusivity values in asymptomatic welders. Toxicol. Sci..

[4] Harischandra DS (2019). Manganese-induced neurotoxicity: New insights into the triad of protein misfolding, mitochondrial impairment, and neuroinflammation. Front. Neurosci..

[5] Egbuna C (2021). Toxicity of nanoparticles in biomedical application: Nanotoxicology. J. Toxicol..

[6] Khanna P, Ong C, Bay BH, Baeg GH (2015). Nanotoxicity: An interplay of oxidative stress, inflammation and cell death. Nanomaterials.

[7] Sobanska Z, Roszak J, Kowalczyk K, Stepnik M (2021). Applications and biological activity of nanoparticles of manganese and manganese oxides in in vitro and in vivo models. Nanomaterials.

[8] Nel A, Xia T, Mädler L, Li N (2006). Toxic potential of materials at the nanolevel. Science.

[9] Cai X (2019). Manganese oxide nanoparticles as MRI contrast agents in tumor multimodal imaging and therapy. Int. J. Nanomed..

[10] Ding B, Zheng P, Ma P, Lin J (2020). Manganese oxide nanomaterials: Synthesis, properties, and theranostic applications. Adv. Mater..

[11] Singh SP (2013). Toxicity assessment of manganese oxide micro and nanoparticles in Wistar rats after 28 days of repeated oral exposure. J. Appl. Toxicol..

[12] Meeker RB, Williams K, Killebrew DA, Hudson LC (2012). Cell trafficking through the choroid plexus. Cell Adher. Migr..

[13] Oszlánczi G (2010). Functional neurotoxicity of Mn-containing nanoparticles in rats. Ecotoxicol. Environ. Saf..

[14] Sadeghi L, Babadi VY, Tanwir F (2018). Manganese dioxide nanoparticle induces Parkinson like neurobehavioral abnormalities in rats. Bratisl Lek Listy.

[15] Quintela T (2013). Analysis of the effects of sex hormone background on the rat choroid plexus transcriptome by cDNA microarrays. PLoS ONE.

[16] Kaur C, Rathnasamy G, Ling EA (2016). The choroid plexus in healthy and diseased brain. J. Neuropathol. Exp. Neurol..

[17] Kratzer I, Ek J, Stolp H (2020). The molecular anatomy and functions of the choroid plexus in healthy and diseased brain. Biochim. Biophys. Acta Biomembr..

[18] Herve F, Ghinea N, Scherrmann JM (2008). CNS delivery via adsorptive transcytosis. AAPS J..

[19] Wang X, Miller DS, Zheng W (2008). Intracellular localization and subsequent redistribution of metal transporters in a rat choroid plexus model following exposure to manganese or iron. Toxicol. Appl. Pharmacol..

[20] Redina OE, Babenko VN (1831). Advances of brain transcriptomics. Genes.

[21] Masocha W, Kombian SB, Edafiogho IO (2016). Evaluation of the antinociceptive activities of enaminone compounds on the formalin and hot plate tests in mice. Sci. Rep..

[22] Miller CK, Halbing AA, Patisaul HB, Meitzen J (2021). Interactions of the estrous cycle, novelty, and light on female and male rat open field locomotor and anxiety-related behaviors. Physiol. Behav..

[23] Miller CK, Krentzel AA, Patisaul HB, Meitzen J (2020). Metabotropic glutamate receptor subtype 5 (mGlu5) is necessary for estradiol mitigation of light-induced anxiety behavior in female rats. Physiol. Behav..

[24] Zhou J (2011). Paeonol increases levels of cortical cytochrome oxidase and vascular actin and improves behavior in a rat model of Alzheimer's disease. Brain Res..

[25] Meng CY (2019). Resveratrol alleviate the injury of mice liver induced by cadmium sulfide nanoparticles. Kaohsiung J. Med. Sci..

[26] Singh SP (2013). Genotoxicity of nano- and micron-sized manganese oxide in rats after acute oral treatment. Mutat. Res..

[27] Kanehisa M, Furumichi M, Sato Y, Ishiguro-Watanabe M, Tanabe M (2021). KEGG: Integrating viruses and cellular organisms. Nucleic Acids Res..

[28] Kanehisa M (2019). Toward understanding the origin and evolution of cellular organisms. Protein Sci..

[29] Hou W (2021). Biocompatible BSA-MnO(2) nanoparticles for in vivo timely permeability imaging of blood-brain barrier and prediction of hemorrhage transformation in acute ischemic stroke. Nanoscale.

[30] Zheng Y, Zhang H, Hu Y, Bai L, Xue J (2018). MnO nanoparticles with potential application in magnetic resonance imaging and drug delivery for myocardial infarction. Int. J. Nanomed..

[31] Tootoonchi MH, Hashempour M, Blackwelder PL, Fraker CA (2017). Manganese oxide particles as cytoprotective, oxygen generating agents. Acta Biomater..

[32] Kumar S, Adjei IM, Brown SB, Liseth O, Sharma B (2019). Manganese dioxide nanoparticles protect cartilage from inflammation-induced oxidative stress. Biomaterials.

[33] Hafez AA, Naserzadeh P, Ashtari K, Mortazavian AM, Salimi A (2018). Protection of manganese oxide nanoparticles-induced liver and kidney damage by vitamin D. Regul. Toxicol. Pharmacol..

[34] Zaitseva NV, Zemlyanova MA, Zvezdin VN, Akafieva TI, Saenko EV (2015). Acute inhalation toxicity of manganese oxide nanoparticles. Nanotechnol. Russ..

[35] Máté Z (2016). Size-dependent toxicity differences of intratracheally instilled manganese oxide nanoparticles: Conclusions of a subacute animal experiment. Biol. Trace Elem. Res..

[36] Zheng W (2022). Choroid plexus-selective inactivation of adenosine A(2A) receptors protects against T cell infiltration and experimental autoimmune encephalomyelitis. J. Neuroinflamm..

[37] Schmitt C, Strazielle N, Richaud P, Bouron A, Ghersi-Egea JF (2011). Active transport at the blood-CSF barrier contributes to manganese influx into the brain. J. Neurochem..

[38] Hai-ming J (2011). Screening identification and Go annotated analysis of manganese-related differentially expressed proteins in the choroid plexus of rats. J. Toxicol..

[39] Zheng W (2001). Toxicology of choroid plexus: Special reference to metal-induced neurotoxicities. Microsc. Res. Tech..

[40] Bornhorst J (2012). Impact of manganese on and transfer across blood-brain and blood-cerebrospinal fluid barrier in vitro. J. Biol. Chem..

[41] Haoui M, Petersen NT, Bjorkgren I, Chung DH, Lishko PV (2021). Choroid plexus epithelial cells as a model to study nongenomic steroid signaling and its effect on ion channel function. Method Enzymol..

[42] Jenkitkasemwong S (2018). SLC39A14 deficiency alters manganese homeostasis and excretion resulting in brain manganese accumulation and motor deficits in mice. Proc. Natl. Acad. Sci. USA.

[43] Wang W, Li S, Wang X, Wang J, Zhang Y (2023). PbO nanoparticles increase the expression of ICAM-1 and VCAM-1 by increasing reactive oxygen species production in choroid plexus. Environ. Sci. Pollut. Res. Int..

[44] Fu X (2014). Regulation of copper transport crossing brain barrier systems by Cu-ATPases: Effect of manganese exposure. Toxicol. Sci..

[45] Limbach LK (2007). Exposure of engineered nanoparticles to human lung epithelial cells: Influence of chemical composition and catalytic activity on oxidative stress. Environ. Sci. Technol..

[46] Mingyan X, Rui M, Zhuolu H, Xinyi M, Jingwen W, Mengxiang C, Chunyan M, Tiesheng H (2022). Neurotoxicity of nano manganese and its effects on choroid plexus-related gene transcription in rats. Asian J. Ecotoxicol..

[47] Nyarko-Danquah I (2020). Manganese accumulation in the brain via various transporters and its neurotoxicity mechanisms. Molecules.

[48] Lin LS (2018). Simultaneous fenton-like ion delivery and glutathione depletion by MnO(2)-based nanoagent to enhance chemodynamic therapy. Angew. Chem..

[49] Gao H (2023). Multifunctional nanomedicines-enabled chemodynamic-synergized multimodal tumor therapy via Fenton and Fenton-like reactions. Theranostics.

[50] Dlamini WW, Nelson G, Nielsen SS, Racette BA (2020). Manganese exposure, parkinsonian signs, and quality of life in South African mine workers. Am. J. Ind. Med..

[51] Browning CL, Green A, Gray EP, Hurt R, Kane AB (2021). Manganese dioxide nanosheets induce mitochondrial toxicity in fish gill epithelial cells. Nanotoxicology.

[52] Gray EP (2020). Chemical and colloidal dynamics of MnO(2) nanosheets in biological media relevant for nanosafety assessment. Small.

