# Author Correction: Transcriptomics-based investigation of manganese dioxide nanoparticle toxicity in rats’ choroid plexus

**DOI:** 10.1038/s41598-024-59889-5

**Published:** 2024-04-23

**Authors:** Chun-Yan Meng, Xin-Yi Ma, Ming-Yan Xu, Sheng-Fei Pei, Yang Liu, Zhuo-Lu Hao, Qing-Zhao Li, Fu-Min Feng

**Affiliations:** 1 School of Public Health, North China University of Science and Technology, Tangshan, Hebei 063210 People’s Republic of China; 2 School of Public Health, Nanjing Medical University, Nanjing, 211166 People’s Republic of China; 3 College of Life Sciences, North China University of Science and Technology, Tangshan, Hebei 063210 People’s Republic of China

Correction to: *Scientific Reports* 10.1038/s41598-023-35341-y, published online 25 May 2023

The original version of this Article contained an error in the spelling of the author **Chun-Yan Meng** which was incorrectly given as **Chun-Yan Memg**.

The original Article has been corrected.

