# Supplementary material for: Transcriptomics-based investigation of manganese dioxide nanoparticle toxicity in rats’ choroid plexus
Source: Sci Rep. 2023 May 25;13:8510. doi: 10.1038/s41598-023-35341-y (PMC10213021; doi:10.1038/s41598-023-35341-y)
Supplement: Supplementary file 1 — Supplementary Information. [file 41598_2023_35341_MOESM1_ESM.docx]

**Supplementary Figures**

**
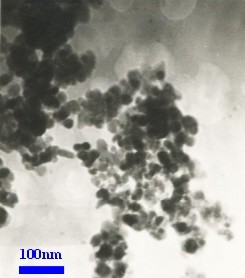
**

**Supplementary Figure 1** Representative electron microscopy image of the synthesized MnO_2_-NP.

B

A


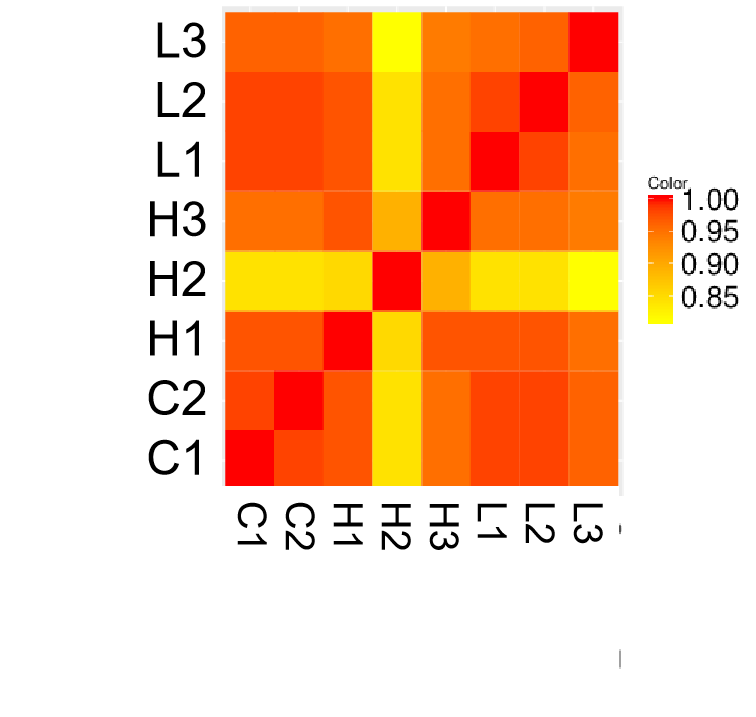

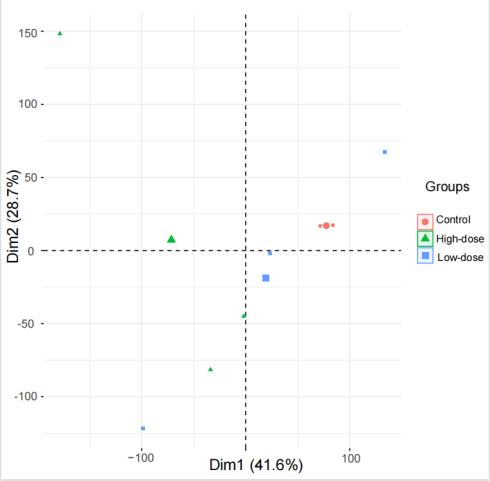


**Supplementary Figure 2** MnO_2_-NP induce a marked change in gene expression in choroid plexus.

1. Principal component analysis and (B) correlation analysis of the CP RNA sequencing data showed the dispersion of treatment (MnO_2_-NP) and control groups. Notes to the images: C1 and C2 belonged to control group.

The chart was drown by an online software (https://cloud.keyandaydayup.com/).

**Supplementary Figure 3** Mn concentration in serum, cerebrospinal fluid, and brain

The levels of Mn in serum, brain and CSF were significantly increased at high dosage of MnO_2_-NP treated.

* represents significant differences compared to control group.

Samples were digested detected by ICP-MS. Brain samples were digested by microwave digestion method in HNO_3._ Serum and CSF samples were obtained directly by digestion with 5% HNO_3_.

**Supplementary Tables**

**Supplementary Table 1** The primer sequences used in this paper

| Gene name | primer sequences | | Product length |
| --- | --- | --- | --- |
| Brinp1 | F | 5' -AATCTGCCAGATGCGAAACA- 3' | 345 |
|  | R | 5' -CTCAGGCTATACCCGAACACC-3' |  |
| Crmp1 | F | 5'-AGAGGCGGACGCATCATCAA -3' | 351 |
|  | R | 5'-CGAATAGTCACAGCAGGATTTGGT-3' |  |
| Synpr | F | 5'-ACTGGCTGGCATTCCTC-3' | 116 |
|  | R | 5'-CTGTACCCACCACTTGACC-3' |  |
| GAPDH | F | 5'- GTTGTGGCTCTGACATGCT -3' | 101 |
|  | R | 5'- CCCAGGATGCCCTTTAGT -3' |  |

**Supplementary Table 2** Statistical results of the raw sequence and the preprocessed sequence

| **Sample** | **Raw reads** | **Raw bases** | **Clean reads** | **Clean bases** | **Average length** | **Clean reads %** | **Clean bases %** | **Q20 %** | **Q30 %** |
| --- | --- | --- | --- | --- | --- | --- | --- | --- | --- |
| D1 | 126226168 | 19060151368 | 124246354 | 17883624609 | 143.9368161 | 98.43% | 93.83% | 98.40% | 95.10% |
| D2 | 122746164 | 18534670764 | 120794618 | 17458456618 | 144.5300867 | 98.41% | 94.19% | 98.35% | 95.05% |
| H1 | 83547890 | 12615731390 | 82210006 | 11899545890 | 144.7457125 | 98.40% | 94.32% | 98.40% | 95.10% |
| H2 | 85799562 | 12955733862 | 84340980 | 12116356184 | 143.6591819 | 98.30% | 93.52% | 98.25% | 94.85% |
| H3 | 102225636 | 15436071036 | 100904616 | 14518791768 | 143.8862992 | 98.71% | 94.06% | 98.40% | 95.05% |
| M1 | 102270176 | 15442796576 | 100602854 | 14484483491 | 143.9768646 | 98.37% | 93.79% | 98.40% | 95.10% |
| M2 | 139219250 | 21022106750 | 136968216 | 19713555412 | 143.9279563 | 98.38% | 93.78% | 98.30% | 94.80% |
| M3 | 105142706 | 15876548606 | 103489380 | 14702678850 | 142.0694457 | 98.43% | 92.61% | 98.45% | 95.25% |

**Supplementary Table 3** Map results of the raw sequence

| **Sample** | **Total clean reads** | **Total mapped** | **Mapped ratio(%)** | **Multiple mapped** | **Unique mapped** | **Read-1** | **Read-2** | **Reads map to '+'** | **Reads map to '-'** | **Non-Splice reads** | **Splice reads** | **Reads Proper pair** |
| --- | --- | --- | --- | --- | --- | --- | --- | --- | --- | --- | --- | --- |
| D1 | 124246354 | 120143535 | 96.70% | 11268192 | 108875343 | 54443778 | 54431565 | 54439044 | 54436299 | 60565862 | 48309481 | 108863076 |
| D2 | 120794618 | 116984920 | 96.80% | 10276314 | 106708606 | 53359264 | 53349342 | 53355310 | 53353296 | 58749840 | 47958766 | 106698630 |
| H1 | 82210006 | 80274795 | 97.60% | 4640145 | 75634650 | 37819599 | 37815051 | 37817740 | 37816910 | 40988744 | 34645906 | 75630054 |
| H2 | 84340980 | 82052691 | 97.30% | 5673954 | 76378737 | 38191415 | 38187322 | 38189007 | 38189730 | 56487790 | 19890947 | 76374626 |
| H3 | 100904616 | 98702920 | 97.80% | 6195327 | 92507593 | 46256242 | 46251351 | 46254299 | 46253294 | 51525292 | 40982301 | 92502648 |
| M1 | 100602854 | 97617246 | 97% | 7978479 | 89638767 | 44821937 | 44816830 | 44819768 | 44818999 | 49686267 | 39952500 | 89633610 |
| M2 | 136968216 | 133021413 | 97.10% | 10833161 | 122188252 | 61097221 | 61091031 | 61094595 | 61093657 | 69090375 | 53097877 | 122182012 |
| M3 | 103489380 | 97736116 | 94.40% | 32046665 | 65689451 | 32846311 | 32843140 | 32845082 | 32844369 | 40001435 | 25688016 | 65686164 |

**Supplementary Table 4** The common differentially expressed genes in each group compared in pairs

| Gene_ID | Symbol | Description | Molecular_function (part) |
| --- | --- | --- | --- |
| ENSRNOG00000026577 | Cpne4 | copine 4 | --- |
| ENSRNOG00000005561 | Brinp1 | BMP/retinoic acid inducible neural specific 1 | negative regulation of mitotic cell cycle,response to chemical stimulus, |
| ENSRNOG00000004781 | Crmp1 | collapsin response mediator protein 1 | cytoskeletal protein binding,protein binding,hydrolase activity, acting on carbon-nitrogen (but not peptide) bonds,binding,catalytic activity，phosphoprotein binding,filamin binding,hydrolase activity |
| ENSRNOG00000049882 | Adcyap1 | adenylate cyclase activating polypeptide 1 receptor type 1 | transferase activity,receptor signaling protein activity,signal transducer activity, protein binding,pyrophosphatase activity,receptor binding,pituitary adenylate cyclase-activating polypeptide receptor binding,hormone activity,G-protein coupled receptor binding,molecular transducer activity,phosphotransferase activity, alcohol group as acceptor, transferase activity, transferring phosphorus-containing groups,kinase activity, peptide hormone receptor binding, GTPase activity |
| ENSRNOG00000038291 | Lrrc63 | leucine rich repeat containing 63 | --- |
| ENSRNOG00000052745 | Met | MET proto-oncogene, receptor tyrosine kinase | transferase activity,receptor signaling protein activity,purine nucleotide binding,signal transducer activity,purine ribonucleoside triphosphate binding,protein heterodimerization activity,protein tyrosine kinase activity, hepatocyte growth factor-activated receptor activity, protein binding,protein dimerization activity, protein complex binding,small molecule binding,heterocyclic compound binding |
| ENSRNOG00000042536 | Pde4d | phosphodiesterase 4D | purine nucleotide binding,ion transmembrane transporter activity,cation channel activity,ryanodine-sensitive calcium-release channel activity,small conjugating protein ligase binding,protein binding,protein domain specific binding,SH3 domain binding,ion channel binding,ligand-gated channel activity,passive transmembrane transporter activity,purine ribonucleotide binding, ion gated channel activity |
| ENSRNOG00000008203 | Synpr | synaptoporin | clathrin-coated vesicle，membrane-bounded vesicle--- |
| ENSRNOG00000007625 | B3galt1 | Beta-1,3-galactosyltransferase 1 | transferase activity,transferase activity, transferring glycosyl groups,UDP-galactose:beta-N-acetylglucosamine beta-1,3-galactosyltransferase activity,beta-1,3-galactosyltransferase activity,UDP-galactosyltransferase activity,UDP-glycosyltransferase activity,catalytic activity,transferase activity, transferring hexosyl groups,galactosyltransferase activity |
| ENSRNOG00000001851 | Far2 | fatty acyl CoA reductase 2 | catalytic activity,oxidoreductase activity, acting on the aldehyde or oxo group of donors,oxidoreductase activity, acting on the aldehyde or oxo group of donors, NAD or NADP as acceptor,fatty-acyl-CoA reductase (alcohol-forming) activity,oxidoreductase activity |
| ENSRNOG00000047545 | Adra2a | adrenoceptor alpha 2A | thioesterase binding,epinephrine binding,transferase activity,receptor signaling protein activity,signal transducer activity,protein heterodimerization activity,protein tyrosine kinase activity,protein homodimerization activity,protein binding,protein dimerization activity,protein complex binding,adrenergic receptor activity,alpha-2C adrenergic receptor binding,transmembrane signaling receptor activity,receptor binding,binding,G-protein coupled receptor binding,alpha-adrenergic receptor activity,molecular transducer activity,protein serine |
| ENSRNOG00000011803 | Lrfn2 | leucine rich repeat and fibronectin type III domain containing 2 | protein binding,binding |
| ENSRNOG00000006631 | Sema3e | semaphorin 3E | protein binding,receptor binding,semaphorin receptor binding |
| ENSRNOG00000014149 | Npy1r | neuropeptide Y receptor Y1 | signal transducer activity,pancreatic polypeptide receptor activity,G-protein coupled peptide receptor activity,transmembrane signaling receptor activity,molecular transducer activity,G-protein coupled receptor activity,neurotransmitter receptor activity,peptide YY receptor activity,neuropeptide receptor activity,peptide receptor activity,receptor activity,signaling receptor activity,neuropeptide Y receptor activity |
| ENSRNOG00000030880 | Hs6st2 | heparan sulfate 6-O-sulfotransferase 2 | transferase activity,heparan sulfate 6-O-sulfotransferase activity,heparan sulfate sulfotransferase activity,catalytic activity,transferase activity, transferring sulfur-containing groups,sulfotransferase activity |
| ENSRNOG00000004828 | Acvr1c | activin A receptor type 1C | transmembrane receptor protein serine/threonine kinase binding,transferase activity,receptor signaling protein activity,purine nucleotide binding,activin receptor activity, type I,signal transducer activity,receptor serine/threonine kinase binding,activin binding,purine ribonucleoside triphosphate binding,transmembrane receptor protein serine,threonine kinase activity,protein binding,SMAD binding,protein complex binding,small molecule binding |
| ENSRNOG00000042446 | Ankrd63 | ankyrin repeat domain 63 | --- |

**Supplementary** **Table 5** Top 40 DEGs regulated by MnO_2_-NP exposure in low dose group comparing with control group (FC=fold change)

| **Down-regulated genes** |  |  |  |  |
| --- | --- | --- | --- | --- |
| **Gene_ID** | **Symbol** | **Description** | **FC** | **p-value** |
| ENSRNOG00000061099 | AABR07033324.1 | --- | 12.31339374 | 0.000 |
| ENSRNOG00000060587 | AABR07002627.1 | --- | 9.305429389 | 7.60E-84 |
| ENSRNOG00000030880 | Hs6st2 | heparan sulfate 6-O-sulfotransferase 2 | 4.167222171 | 0.001518518 |
| ENSRNOG00000053741 | AABR07052664.1 | --- | 3.947106672 | 0.001721431 |
| ENSRNOG00000002974 | Cdx4 | caudal type homeo box 4 | 3.94042483 | 2.98E-18 |
| ENSRNOG00000043048 | Zfp951 | zinc finger protein 951 | 3.890648484 | 0.001718326 |
| ENSRNOG00000016737 | Tcerg1l | transcription elongation regulator 1-like | 3.795305123 | 0.002142793 |
| ENSRNOG00000055672 | Gpx2 | glutathione peroxidase 2 | 3.561152418 | 0.004011436 |
| ENSRNOG00000058727 | LOC100910284 | brevican core protein-like | 3.555716177 | 0.004342707 |
| ENSRNOG00000034038 | AABR07005844.1 | --- | 3.532789261 | 0.016455306 |
| ENSRNOG00000033010 | Akr1c12 | aldo-keto reductase family 1, member C12 | 3.385674681 | 0.007334784 |
| ENSRNOG00000042446 | Ankrd63 | ankyrin repeat domain 63 | 3.33071965 | 0.012112562 |
| ENSRNOG00000057619 | LOC100912068 | hypermethylated in cancer 1 protein-like | 3.31246724 | 0.040461023 |
| ENSRNOG00000002960 | LOC100909913 | norrin-like | 3.263520668 | 0.01746729 |
| ENSRNOG00000019615 | Colq | collagen like tail subunit of asymmetric acetylcholinesterase | 3.216292969 | 0.012627547 |
| ENSRNOG00000004828 | Acvr1c | activin A receptor type 1C | 3.018312608 | 0.02285569 |
| ENSRNOG00000021256 | Adra1d | adrenoceptor alpha 1D | 2.957720636 | 0.024126032 |
| ENSRNOG00000012259 | Il22ra2 | interleukin 22 receptor subunit alpha 2 | 2.875456329 | 0.029380291 |
| ENSRNOG00000053400 | Cfi | complement factor I | 2.858559748 | 0.032669487 |
| ENSRNOG00000008203 | Synpr | synaptoporin | 2.821171946 | 0.014779608 |
|  | | | | |
| **Up-regulated genes** | | | | |
| **Gene_ID** | **Symbol** | **Description** | **FC** | **p-value** |
| ENSRNOG00000050024 | Ms4a4a | membrane-spanning 4-domains, subfamily A, member 4A | 5.069027117 | 2.04E-05 |
| ENSRNOG00000045611 | LOC100911403 | membrane-spanning 4-domains subfamily A member 4A-like | 5.000959669 | 4.54E-05 |
| ENSRNOG00000016632 | Dsg3 | desmoglein 3 | 4.661080731 | 0.000553207 |
| ENSRNOG00000046151 | Tubb1 | tubulin, beta 1 class VI | 4.560070183 | 0.010951516 |
| ENSRNOG00000049513 | Lenep | lens epithelial protein | 4.425290329 | 0.033683404 |
| ENSRNOG00000016283 | Meig1 | meiosis/spermiogenesis associated 1 | 3.931468272 | 0.021753802 |
| ENSRNOG00000020013 | Psrc1 | proline and serine rich coiled-coil 1 | 3.751428597 | 0.017940129 |
| ENSRNOG00000000055 | Fcrl6 | Fc receptor-like 6 | 3.731221468 | 0.0472922 |
| ENSRNOG00000048321 | Tnfsf8 | tumor necrosis factor superfamily member 8 | 3.730450882 | 0.006753581 |
| ENSRNOG00000011823 | Tfap2b | transcription factor AP-2 beta | 3.581103867 | 0.006346151 |
| ENSRNOG00000058538 | AABR07002001.1 | --- | 3.551669144 | 0.014051746 |
| ENSRNOG00000018268 | Hhip | Hedgehog-interacting protein | 3.490074928 | 0.006803986 |
| ENSRNOG00000051163 | Rn50_10_0705.2 | --- | 3.455213051 | 0.014677472 |
| ENSRNOG00000026051 | RGD1562652 | similar to class I histocompatibility antigen alpha chain - cotton-top tamarin | 3.400260167 | 0.016998886 |
| ENSRNOG00000038291 | Lrrc63 | leucine rich repeat containing 63 | 3.358171129 | 0.016346965 |
| ENSRNOG00000001360 | Stag3 | stromal antigen 3 | 3.339459268 | 0.006333898 |
| ENSRNOG00000052745 | Met | MET proto-oncogene, receptor tyrosine kinase | 3.099918483 | 0.038039081 |
| ENSRNOG00000049882 | Adcyap1 | adenylate cyclase activating polypeptide 1 | 3.031585659 | 0.011560188 |
| ENSRNOG00000054174 | AABR07030351.1 | --- | 3.010918428 | 0.042483141 |
| ENSRNOG00000022099 | Trim72 | tripartite motif containing 72 | 2.859565499 | 0.029627773 |

**Supplementary Table 6** Top 40 DEGs regulated by MnO_2_-NP exposure in high dose group comparing with control group (FC=fold change)

| **Down-regulated genes** | | | | |
| --- | --- | --- | --- | --- |
| **Gene_ID** | **Symbol** | **Description** | **FC** | **p-value** |
| ENSRNOG00000054945 | LOC257642 | rRNA promoter binding protein | 16.18836527 | 9.81E-90 |
| ENSRNOG00000061099 | AABR07033324.1 | --- | 12.31339374 | 2.02E-47 |
| ENSRNOG00000053717 | Metazoa_SRP | Metazoan signal recognition particle RNA | 7.669374865 | 1.67E-15 |
| ENSRNOG00000052862 | Metazoa_SRP | Metazoan signal recognition particle RNA | 7.669374865 | 1.67E-15 |
| ENSRNOG00000060381 | Col15a1 | collagen type XV alpha 1 chain | 6.690827968 | 3.52E-06 |
| ENSRNOG00000047594 | Heatr1 | HEAT repeat containing 1 | 6.607132116 | 3.46E-05 |
| ENSRNOG00000060518 | LOC257642 | rRNA promoter binding protein | 5.715476658 | 5.67E-13 |
| ENSRNOG00000045586 | AABR07073186.1 | --- | 4.731416994 | 1.56E-09 |
| ENSRNOG00000057732 | AABR07043288.1 | --- | 4.462054683 | 1.73E-05 |
| ENSRNOG00000015403 | Cd52 | CD52 molecule | 4.437051935 | 0.035138146 |
| ENSRNOG00000046600 | AABR07015066.1 | --- | 4.40250567 | 1.34E-10 |
| ENSRNOG00000055956 | AABR07015078.1 | --- | 4.399245397 | 1.98E-10 |
| ENSRNOG00000060896 | AABR07063424.1 | rRNA promoter binding protein | 4.377031973 | 9.21E-11 |
| ENSRNOG00000009436 | Hemgn | hemogen | 4.300001581 | 5.33E-06 |
| ENSRNOG00000000167 | Alas2 | 5-aminolevulinate synthase 2 | 4.287629379 | 0.013750417 |
| ENSRNOG00000059504 | AABR07015078.2 | --- | 4.272839287 | 8.05E-08 |
| ENSRNOG00000050156 | AABR07063425.2 | --- | 4.265507664 | 8.42E-09 |
| ENSRNOG00000055154 | AABR07063462.1 | --- | 4.259983217 | 1.47E-08 |
| ENSRNOG00000046707 | AABR07063425.1 | --- | 4.25531275 | 2.12E-08 |
| ENSRNOG00000054657 | AABR07015067.1 | --- | 4.253582827 | 1.74E-08 |
|  | | | | |
| **Up-regulated genes** | | | | |
| **Gene_ID** | **Symbol** | **Description** | **FC** | **p-value** |
| ENSRNOG00000045554 | LOC102551901 | protein lifeguard 2-like | 8.546302141 | 0.005813188 |
| ENSRNOG00000050450 | LOC100911951 | Kv channel-interacting protein 2-like | 8.52610286 | 0.001029422 |
| ENSRNOG00000049495 | Krt71 | keratin 71 | 7.498461595 | 7.78E-07 |
| ENSRNOG00000014503 | Celf4 | CUGBP, Elav-like family member 4 | 7.469343878 | 5.25E-06 |
| ENSRNOG00000052022 | Pnma3 | paraneoplastic Ma antigen 3 | 7.426687014 | 2.45E-06 |
| ENSRNOG00000014006 | Neto1 | neuropilin and tolloid like 1 | 7.365096223 | 8.80E-10 |
| ENSRNOG00000030840 | Cadm2 | cell adhesion molecule 2 | 7.353902336 | 1.40E-05 |
| ENSRNOG00000002911 | Alb | albumin | 7.263819213 | 2.63E-08 |
| ENSRNOG00000004372 | Cbln4 | cerebellin 4 precursor | 7.203168855 | 2.51E-09 |
| ENSRNOG00000040166 | Ankrd34b | ankyrin repeat domain 34B | 7.161009154 | 1.49E-05 |
| ENSRNOG00000017346 | Myt1 | myelin transcription factor 1 | 7.120203611 | 9.26E-06 |
| ENSRNOG00000018690 | Rgs17 | regulator of G-protein signaling 17 | 7.070016822 | 3.33E-08 |
| ENSRNOG00000002349 | Gabra2 | gamma-aminobutyric acid type A receptor alpha2 subunit | 7.069001264 | 0.032866881 |
| ENSRNOG00000023601 | Elavl4 | ELAV like RNA binding protein 4 | 7.055046622 | 4.03E-09 |
| ENSRNOG00000008790 | Gdap1l1 | ganglioside-induced differentiation-associated protein 1-like 1 | 6.926162416 | 2.40E-05 |
| ENSRNOG00000000693 | Svop | SV2 related protein | 6.924983198 | 5.35E-09 |
| ENSRNOG00000037476 | Galnt9 | polypeptide N-acetylgalactosaminyltransferase 9 | 6.860249303 | 7.60E-06 |
| ENSRNOG00000009209 | Slitrk1 | SLIT and NTRK-like family, member 1 | 6.827442288 | 0.048448734 |
| ENSRNOG00000014290 | Grm1 | glutamate metabotropic receptor 1 | 6.813318202 | 9.57E-06 |
| ENSRNOG00000016091 | Tmem169 | transmembrane protein 169 | 6.808465027 | 1.58E-07 |

**Supplementary Table 7** Top 40 DEGs regulated by MnO_2_-NP exposure in high dose group comparing with low dose group (FC=fold change)

| **Down-regulated genes** | | | | |
| --- | --- | --- | --- | --- |
| **Gene_ID** | **Symbol** | **description** | **FC** | **p-value** |
| ENSRNOG00000054945 | LOC257642 | rRNA promoter binding protein | 15.89620907 | 1.37E-06 |
| ENSRNOG00000053717 | Metazoa_SRP | Metazoan signal recognition particle RNA | 8.437341744 | 7.87E-07 |
| ENSRNOG00000052862 | Metazoa_SRP | Metazoan signal recognition particle RNA | 8.437341744 | 7.87E-07 |
| ENSRNOG00000060518 | LOC257642 | rRNA promoter binding protein | 5.957565737 | 5.48E-10 |
| ENSRNOG00000000167 | Alas2 | 5-aminolevulinate synthase 2 | 5.591205406 | 0.000687336 |
| ENSRNOG00000015403 | Cd52 | CD52 molecule | 5.374162248 | 0.00019087 |
| ENSRNOG00000009436 | Hemgn | hemogen | 5.1834185 | 1.20E-06 |
| ENSRNOG00000029886 | Hba-a2 | hemoglobin alpha, adult chain 2 | 5.125220379 | 4.67E-06 |
| ENSRNOG00000002776 | Sell | selectin L | 5.082988022 | 0.000483311 |
| ENSRNOG00000046600 | AABR07015066.1 | --- | 5.081379579 | 2.66E-09 |
| ENSRNOG00000055956 | AABR07015078.1 | --- | 5.080769059 | 3.59E-09 |
| ENSRNOG00000047321 | Hba-a2 | hemoglobin alpha, adult chain 2 | 5.066664786 | 2.44E-06 |
| ENSRNOG00000060896 | AABR07063424.1 | rRNA promoter binding protein | 5.055021717 | 2.63E-09 |
| ENSRNOG00000015682 | Kel | Kell blood group, metallo-endopeptidase | 5.001437182 | 0.003855991 |
| ENSRNOG00000050156 | AABR07063425.2 | --- | 4.99873402 | 4.37E-09 |
| ENSRNOG00000055154 | AABR07063462.1 | --- | 4.992853228 | 4.31E-09 |
| ENSRNOG00000046707 | AABR07063425.1 | --- | 4.988111472 | 7.01E-09 |
| ENSRNOG00000054657 | AABR07015067.1 | --- | 4.981198182 | 4.79E-09 |
| ENSRNOG00000050545 | AABR07015056.1 | --- | 4.979934997 | 4.29E-09 |
| ENSRNOG00000059504 | AABR07015078.2 | --- | 4.971745893 | 2.78E-09 |
|  |  |  |  |  |
| **Up-regulated** |  |  |  |  |
| **Gene_ID** | **Symbol** | **Description** | **FC** | **p-value** |
| ENSRNOG00000050450 | LOC100911951 | Kv channel-interacting protein 2-like | 8.790750521 | 7.64E-07 |
| ENSRNOG00000002151 | LOC103693564 | transgelin-3 | 8.640286479 | 0.016153887 |
| ENSRNOG00000045554 | LOC102551901 | protein lifeguard 2-like | 8.546302141 | 3.16E-05 |
| ENSRNOG00000047080 | Gng4 | G protein subunit gamma 4 | 7.87348964 | 0.049501169 |
| ENSRNOG00000047635 | Tmem178b | transmembrane protein 178B | 7.668146844 | 0.009697313 |
| ENSRNOG00000049495 | Krt71 | keratin 71 | 7.498461595 | 1.89E-11 |
| ENSRNOG00000015393 | Slc32a1 | solute carrier family 32 member 1 | 7.475176688 | 0.046126829 |
| ENSRNOG00000022767 | Elfn1 | extracellular leucine-rich repeat and fibronectin type III domain containing 1 | 7.411392258 | 0.004504786 |
| ENSRNOG00000024712 | Insc | inscuteable homolog (Drosophila) | 7.350909169 | 1.30E-11 |
| ENSRNOG00000004560 | Cacna1b | calcium voltage-gated channel subunit alpha1 B | 7.33664817 | 0.006283877 |
| ENSRNOG00000002349 | Gabra2 | gamma-aminobutyric acid type A receptor alpha2 subunit | 7.288502122 | 0.011497392 |
| ENSRNOG00000014302 | Dlgap3 | DLG associated protein 3 | 7.217794953 | 1.10E-05 |
| ENSRNOG00000021507 | Luzp2 | leucine zipper protein 2 | 7.203734514 | 0.000113514 |
| ENSRNOG00000005669 | Car8 | carbonic anhydrase 8 | 7.006559302 | 1.68E-10 |
| ENSRNOG00000001575 | Grik1 | glutamate ionotropic receptor kainate type subunit 1 | 6.950646021 | 5.78E-10 |
| ENSRNOG00000061939 | LOC100911886 | collagen alpha-2(XI) chain-like | 6.910924567 | 1.81E-10 |
| ENSRNOG00000049802 | AABR07031533.1 | --- | 6.88757378 | 3.29E-10 |
| ENSRNOG00000008203 | Synpr | synaptoporin | 6.819138162 | 8.89E-11 |
| ENSRNOG00000030840 | Cadm2 | cell adhesion molecule 2 | 6.817078434 | 0.000782996 |
| ENSRNOG00000032490 | Cdh7 | cadherin 7 | 6.793092364 | 0.001853205 |
